# Membrane Lipid Phase Transition Behavior of Oocytes from Three Gorgonian Corals in Relation to Chilling Injury

**DOI:** 10.1371/journal.pone.0092812

**Published:** 2014-03-26

**Authors:** Chiahsin Lin, Fu-Wen Kuo, Suchana Chavanich, Voranop Viyakarn

**Affiliations:** 1 National Museum of Marine Biology & Aquarium, Checheng, Pingtung, Taiwan; 2 Institute of Marine Biotechnology, National Dong Hwa University, Checheng, Pingtung, Taiwan; 3 Reef Biology Research Group, Department of Marine Science, Faculty of Science, Chulalongkorn University, Bangkok, Thailand; King's College London, United Kingdom

## Abstract

The lipid phase transition (LPT) from the fluid liquid crystalline phase to the more rigid gel structure phase that occurs upon exposure to low temperatures can affect physical structure and function of cellular membranes. This study set out to investigate the membrane phase behavior of oocytes of three gorgonian corals; *Junceela fragilis, J. juncea* and *Ellisella robusta*,at different developmental stages after exposure to reduced temperatures. Oocytes were chilled to 5°C for 48, 96 or 144 h, and the LPT temperature (LPTT) was determined with Fourier Transform Infrared (FTIR) spectroscopy. The *J. fragilis* oocytes had a higher LPTT (∼23.0–23.7°C) than those of *J. juncea* and *E. robusta* oocytes (approximately 18.3–20.3°C). Upon chilling for 96 h at 5°C, the LPTTs of *J. juncea* and *E. robusta* oocytes in the early (18.0±1.0 and 18.3±0.6°C, respectively) and late (17.3±0.6 and 17.7±1.2°C, respectively) stages were significantly lower than those of *J. fragilis* oocytes (20.3±2.1 and 19.3±1.5°C for the early and late stages, respectively). The LPTTs of early stage gorgonian oocytes was significantly lower than those of late stage oocytes. These results suggest that the LPT of three gorgonian oocytes at different developmental stages may have been influenced by the phospholipid composition of their plasma membranes, which could have implications for their low temperature resistance.

## Introduction

Cryopreservation technology applied to the preservation of coral germ cells has recently shown promise as a potential *ex situ* conservation technique [1]. Low temperature preservation of coral oocytes may ultimate become an essential tool for conservation of genetic resources and regeneration of populations of valuable and endangered coral species, as this life history stage is less susceptible to cryoinjuries [1], [2]. Our previous studies have found that hard coral (*Echinopora* spp.) and gorgonian coral (*Junceella juncea* and *Junceella fragilis*) oocytes had significant levels of chilling tolerance down to 5°C and 0°C; however, these oocytes were susceptible to chilling injuries at sub-zero temperatures. For instance, mitochondrial activities decreased dramatically after four hours of chilling [1], [2].

The microtubules [3], [4], [5], cytoskeletal organization [6], [7], [8], and intracellular organelles [9] of oocytes have all shown to be damaged upon exposure to reduced temperatures. It has been proposed that lipid phase transition (LPT) may have important implications for susceptibility to chilling injury, as LPT can affect cell membrane properties, such as their function and integrity [10], [11], [12]. When cells are chilled below their LPT temperature (LPTT), cell membranes undergo a transition from the liquid to the gel phase [12]. It has been reported that lowering the temperature results in profound changes in the phase transition of lipids of the gametes of various species, and LPT has been found to influence the degree of cryoinjury inpig sperm membranes [13] and shrimp embryos [14], as well as oocytes from humans [12], cattle [10], sheep [15], and zebrafish [16].

Phospholipids are major components of cellular membranes, and the transition from the gel phase to the liquid-crystalline face at low temperatures can cause membrane damage [17]. Our previous studies indicated that phosphatidylethanolamine (PE) and phosphatidylcholine (PC) are the main phospholipids in the membranes of oocytes from two gorgonian coral species; such a high PE and PC content helps to establish a higher surface viscosity and is associated with lower membrane melting temperatures [18]. The fatty acid composition of phospholipids also strongly influences the LPT profile of cellular membranes [15]. For instance, cellular membranes with short chain polyunsaturated fatty acids are more fluid at low temperatures and are more resistant to chilling stress [17], [18], [19], [20].

The fact that a low LPTT is associated with resistance to chilling stress [10], [12] suggests that coral species with greater degrees of cell membrane fluidity will more likely survive the cryopreservation process. Improving our understanding of the LPT mechanism through chilling experiments may therefore help in developing an effective cryopreservation method for chilling-sensitive coral cell types, including oocytes. In this study, we used Fourier transform infrared (FTIR) spectroscopy to investigate the membrane lipid phase behavior of oocytes of different developmental stages from three coral species; *Junceela fragilis, J. juncea*, and *Euplexaura robusta*, after low temperature exposure in order to better understand why these oocytes are so sensitive to dramatic reductions in temperature.

## Results

### LPT of Coral Oocytes

**Figure 1:**
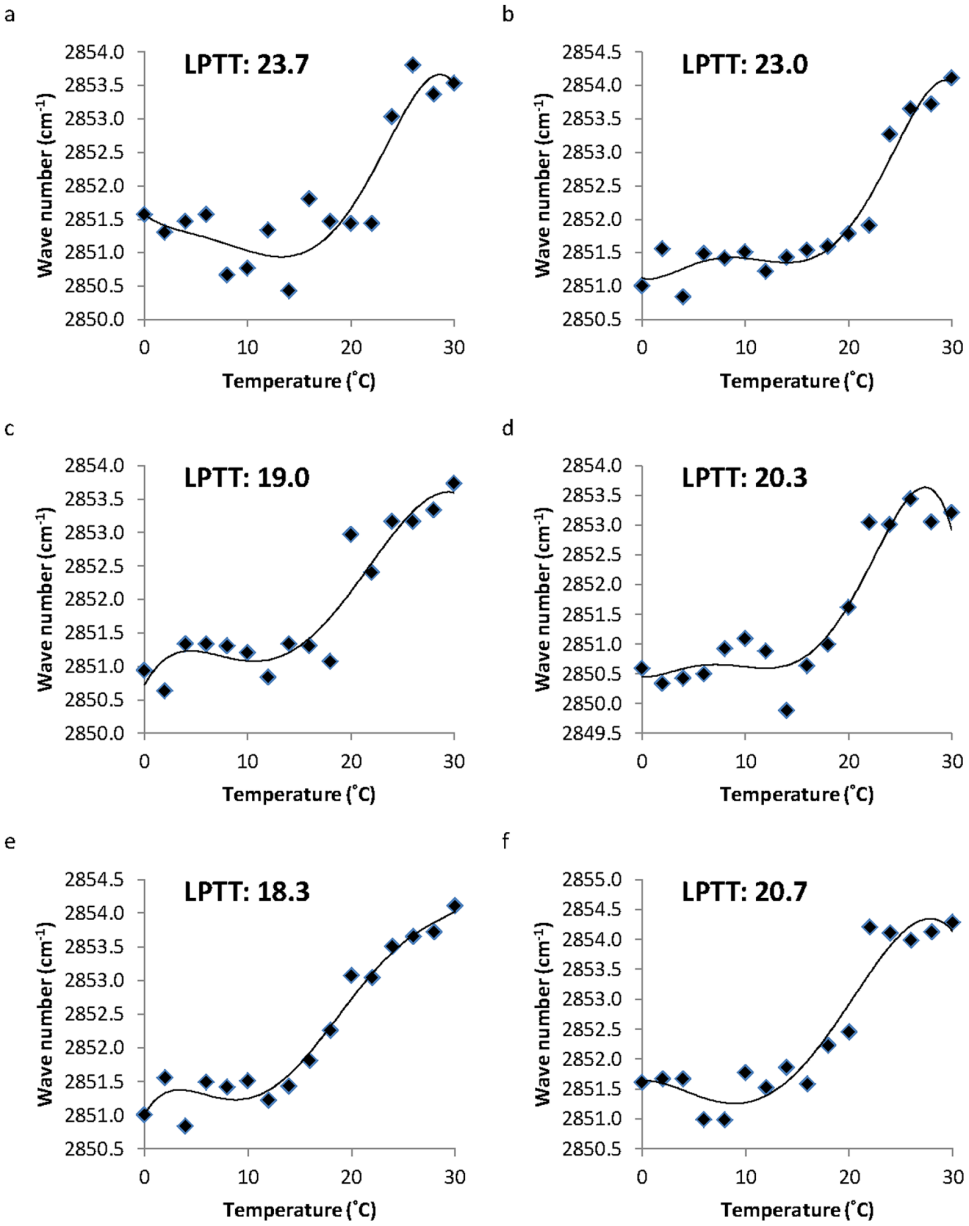
The lipid phase transition of *Juncea fragilis, J. juncea*, and *Ellisella robusta* oocytes at early (1a, c, and e, respectively) and late (1b, d, and f, respectively) stages, as determined from a Fourier Transform Infrared analyzer. The inflection point of the lipid phase transition curve from liquid crystalline to gel phase represents the the lipid phase transition temperature.

The LPT of three gorgonian oocyte membranes from the liquid crystalline to the gel phase revealed a different LPTT at different developmental stages (Fig. 1). The *J. fragilis* oocytes demonstrated LPTTs ranging from 23.0±0.6 to 23.7±0.7°C. In contrast, *J. juncea* and *E. robusta* oocytes were found to have LPTTs of approximately 18.3±0.7 and 20.3±0.7°C, respectively (Fig. 1). The LPT occurred at a significantly lower temperature in the early stage oocytes of *J. juncea* and *E. robusta* (*p<*0.05 for each species), whilst there were no significant differences in the LPTT between the early and late stage oocytes of *J. fragilis* (*p>*0.05, Fig. 1). The membrane LPTT in the early stage oocytes of *J. fragilis, J. juncea*, and *E. robusta* were 23.7±0.7°C, 19±0.6°C, and 18.3±0.7°C (Fig. 1a, 1c, and 1e, respectively), respectively, while the late stage oocyte LPTTs were 23±0.6°C, 20.3±0.7°C, and 20.3±0.3°C, respectively (Fig. 1b, 1d, and 1f, respectively).

### Inter-specific Differences in Coral Oocyte LPT

**Figure 2:**
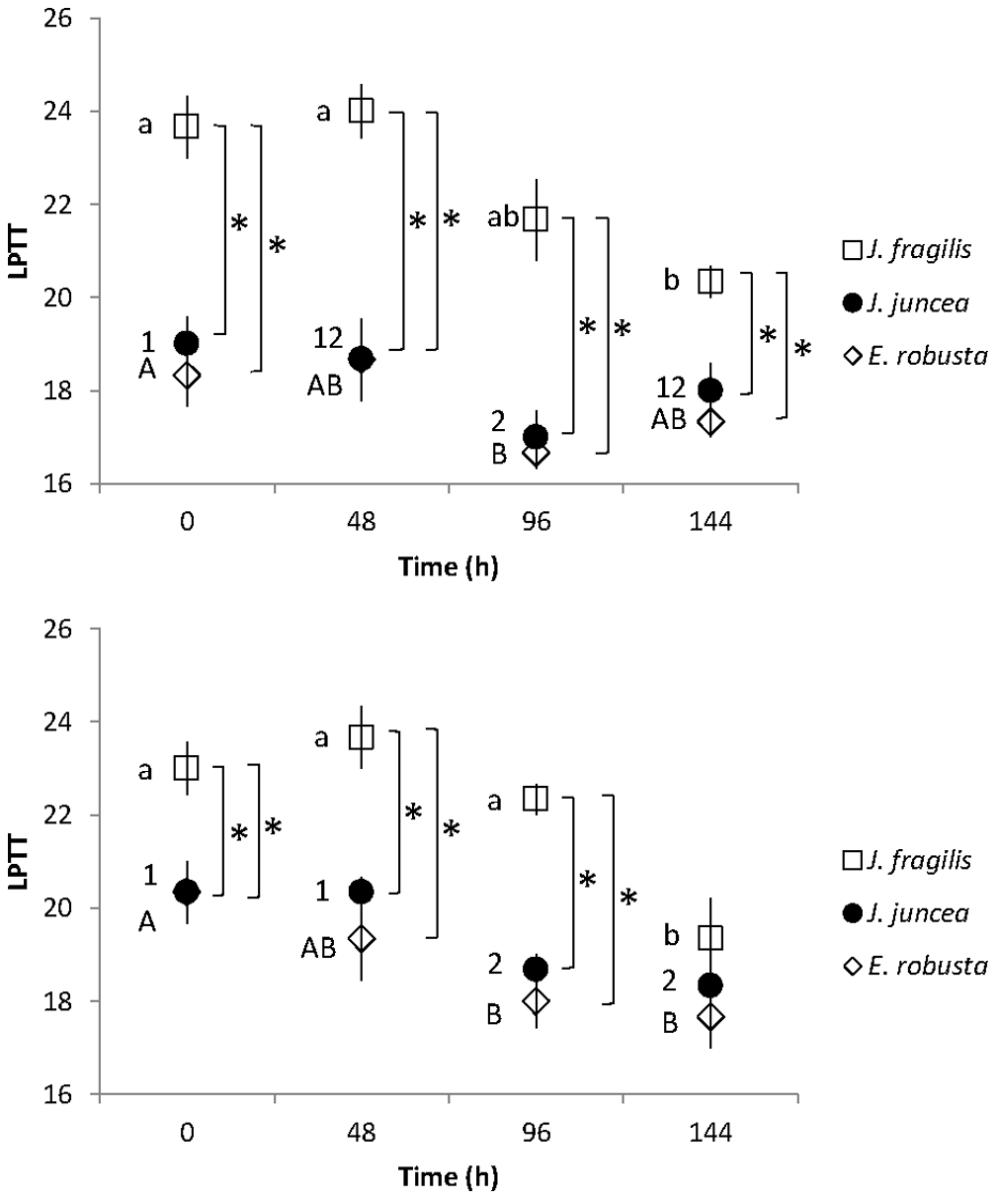
The lipid phase transition temperature (LPTT) of *Juncea fragilis*, *J. juncea*, and *Euplexaura. robusta* oocytes at early (a) and late (b) developmental stages after chilling at 5°C for up to 144 h in filtered seawater. Error bars indicate standard error of the means. Astrices represent significant differences (*p*<0.05) between LPTTs of *J. fragilis, J. juncea*, and *E. robusta* oocytes at the same chilling time period.

The LPTTs of oocyte membranes of *J. juncea* and *E. robusta* were significantly lower after 96 h of exposure to 5°C than those of oocytes of *J. fragilis* (*p<*0.05, Fig. 2a and 2b). The LPTT values were significantly lower for early stage oocytes of *J. juncea* and *E. robusta* at all chilling exposure periods than those of *J. fragilis* oocytes (Fig. 2a). The LPTT in the late stage oocytes of *J. fragilis* was 19.3±1.5°C after chilling at 5°C for 144 h, significantly less than that of controls incubated at 25C for this same length of time (23.0±2.0°C, Tukey’s HSD, *p*<0.05). Membrane LPTTs of *J. juncea* and *E. robusta* late stage oocytes were 18.7±1.2 and 18.0±1.0°C, respectively, after chilling for 96 h (Fig. 2), a decrease of nearly 2°C in comparison to the respective controls (21.0±2.0 and 20.7±1.2°C, respectively).

### Effect of Chilling on the LPT of Coral Oocytes at Different Developmental Stages

**Figure 3:**
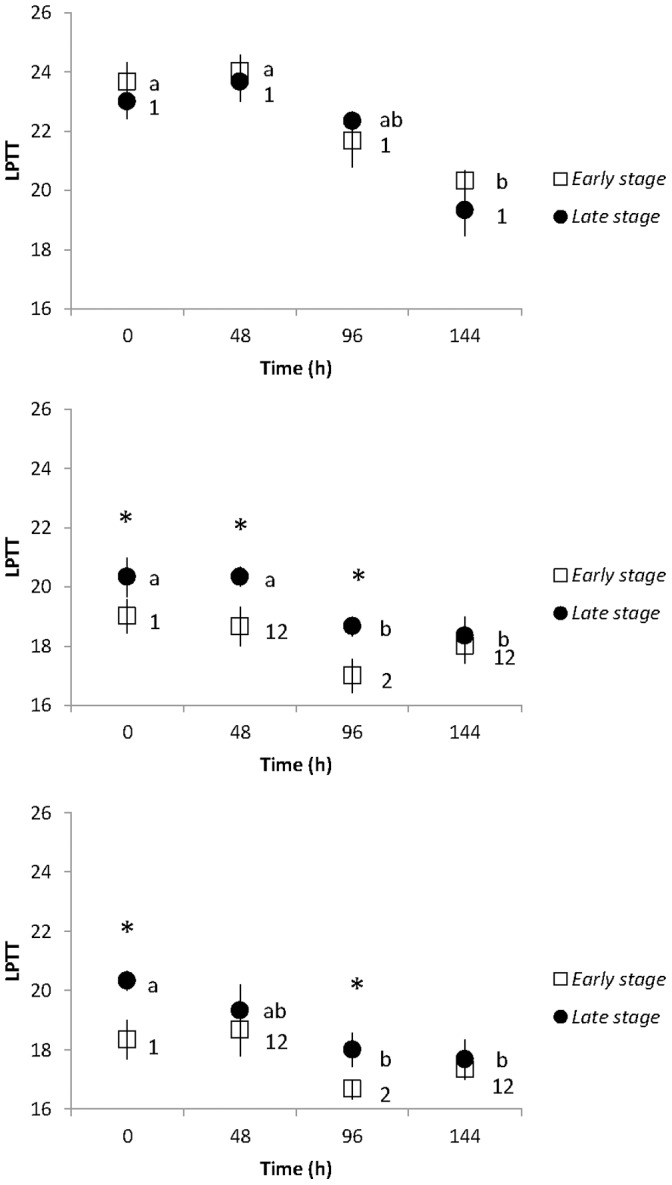
The lipid phase transition temperature (LPTT) of early and late stages oocytes of *Juncea fragilis* (a), *J. juncea* (b), and *Ellisella robusta*. Error bars indicate standard error of the means. Astrices represent significant difference between early and late stage at the same chilling time period (*p<*0.05).

The LPTTs of *J. fragilis, J. juncea*, and *E. robusta* oocytes at different developmental stages were measured after chilling at 5°C for up to 144 h, and the results are shown in Figure 3. The chilled oocytes of *J. juncea* and *E. robusta* had a significantly lower LPTT after 96 h of exposure to5°C than those of control group at all stages (*p<*0.05, Fig. 3b and 3c), whilst LPTTs of oocyte membranes of *J. fragilis* were not significantly lower than time 0 controls until 144 h of chilling exposure had occurred (*p<*0.05, Fig. 3a). The LPTT values of *J. juncea* and *E. robusta* oocytes in the early and late stages were significantly lower (18.0±1.0 and 17.3±0.6°C, 18.3±0.6 and 17.7±1.2°C) after 96 h chilling at 5°C compared to those obtained from *J. fragilis* oocytes (20.3±2.1 and 19.3±1.5°C for early and late stage oocytes, respectively, *p<*0.05). Oocyte membranes of *J. juncea and E. robusta* showed a less well-defined LPTT value in the early and late stages (*p>*0.05, Fig. 3). The lowest LPTT (16.7±0.6°C was obtained in the early stage ooctyes of *E. robusta* after chilling at 5°C for 144 h (Fig. 3c). There were no statistical differences in the LPTTs between the early and late stage oocytes of any of the three coral species at any sampling time (Fig. 3).

## Discussion

Many studies have suggested that chilling damage is a major obstacle for successful cryopreservation of gametes, and cell membranes are thought to be amongst the most sensitive cellular structures to temperature decreases [1], [2], [14], [21], [22]. Chilling damage has been directly correlated with thermal LPT [11], [14], [22]. For instance, the LPT from the fluid liquid crystalline phase to the more rigid gel phase [23] is associated with chilling injury in mammalian sperm [11], [13] and fish oocytes [22]. Species whose lipid membranes demonstratelower LPTTs have typically been found to better resist cold stress [10], [12]. Our current results showed a significant difference between the LPTT values between three gorgonian coral oocytes. The phase transition temperature of *J. fragilis* oocytes was significantly higher than oocytes of *J. juncea* and *E. robusta*, and this may indicate that the former are more prone to chilling injury.

Phospholipids are the major lipid component of cellular membrane, and the fatty acids within the phospholipids strongly influence the membrane properties [12], [24], [25]. Fatty acids are abundant in the phospholipid fraction of oocytes from cattle [24], [26], pigs [27], sheep [28], and some coral species [14], [18], [29], providing a valuable energy source [29], [30], or as precursors for the elongation of long chain fatty acids [31], [32] influencing reproduction and oocyte membrane fluidity [24], [33]. Oocyte membrane fluidity is influenced by temperature alterations between seasons that may be due to changes in fatty acid composition [24], [34], [35]. The LPT is also strongly affected by the fatty acid composition of the membrane [25]. Seasonal studies of bovine oocytes have found that oocyte membranes are composed of more saturated fatty acids during the summer whereas they contain mainly mono- and polyunsaturated fatty acids during the winter months [24]. This resulted in LPTT decreases of 6°C from the summer to the winter [24].

In the ascidian *Halocynthia aurantium,* temperature-induced changes in the phase transition temperature of phospholipids decreased by 10°C in both the summer and winter [34], [35]. In the present study, the LPTT of three gorgonian coral oocytes occurred over a broad temperature range, from 16°C to 23°C, which suggests the presence of a variety of different types of fatty acids in the membranes as well as a high concentration of long chain fatty acids. We have also found that *J. fragilis* oocytes were extremely sensitive to chilling. The LPTT of *J. fragilis* oocytes dropped to 3.5°C lower than controls after chilling at 5°C for 144 h. On the other hand, *J. juncea* and *E. robusta* oocytes showed a lower transition temperature at approximately 2°C under the same condition and yet did not cause damage to the membranes. Presumably, damage was avoided because the *J. juncea* and *E. robusta* oocytes had lower LPTT value, so the injury that would result normally was avoided.

Early studies conducted on the cryosensitivity of late stage oocytes found that the main cellular damage occurred due to meiotic spindle disorganization followed by microtubule depolymerization [36], [37]. Oocytes at later developmental stages are extremely sensitive to chilling temperatures, with the predominant damage having been documented to occur at the plasma membrane [10]. However, immature, early stag oocytes are arrested in prophase of the first meiotic division and do not contain any polymerized microtubules [38], [39], [40]. Our previous study showed that late stage coral oocytes were more susceptible to cryoinjury than early stage oocytes [1]. Early stage *J. juncea* and *E. robusta* oocytes had lower LPTT value than those of *J. fragilis* at all stages, indicating that the microtubular organization of early stage oocytes may be less prone to chilling damage.

The composition of phospholipids in cellular membrane has also been linked to chilling sensitivity of coral oocytes [18], [20]. Our previous studies have suggested that PE and PC are the more abundant phospholipids in early stage oocytes, and they collectively help to create a higher membrane surface viscosity [18], [20]. On the basis of previous reports and our present findings, we suggest that changes in the LPT behavior of coral oocyte membranes of three species at different developmental stages may be a function of changes in phospholipid composition at different stages of oogenesis.

The present study membrane phase behavior of two gorgonian coral oocytes was identified, which induced the lethality after chilling for the oocytes. The present study has showed evidence of changes in oocyte membrane fluidity with depth. In fact, as water pressure increases with increasing depth, temperature decreases more abruptly in deeper water. Our previous studies have demonstrated that the higher concentration of phospholipid fatty acid in *J. juncea* oocytes increased the probability of membrane fluidity with depth [20]. As a result, the oocytes from the three gorgonian coral showed significant differences in LPTT and sensitivity with increasing depth.

The phospholipid fatty acid composition of coral oocyte membranes is influenced by the presence of dinoflagellate endosymbionts (genus *Symbiodinium*) [18]. It was demonstrated that enriching diets with polyunsaturated fatty acid changes the fatty acid profile of ewe oocytes, resulting in a reduction of oocyte chilling susceptibility [15]. Studies on the phase behavior of lipids of marine invertebrate (*H. aurantium*) cells have also found that the change in phase behavior of phospholipids is relevant to the decrease of saturated to polyunsaturated fatty acid ratios [34]. The present study revealed that *J. juncea* and *E. robusta* oocytes had lower LPTTs than oocytes of *J. fragilis*. It is possible that *J. fragilis* oocytes inherit dinoflagellates and so contain less unsaturated fatty acids than *J. juncea* and *E. robusta* oocytes, the latter two of which carry no symbiotic dinoflagellates. Our present study also found that membranes of *J. juncea* and *E. robusta* oocytes exhibited altered physical properties of their membranes and decreased in the LPTT (20.7–16.7°C), whilst *J. fragilis* had a higher (LPTT) value (approximately 23°C); this may indicate that *J. juncea* and *E. robusta* oocytes are more resistance to chilling injury.

Herein the LPT of coral oocytes was reported for the first time, and our findings confirm preliminary results from our previous studies that LPT and chilling sensitivity are associated with the phospholipid composition of the membrane [18], [20]. In this study on the LPT of gorgonian oocytes, higher LPTT values were observed in *J. juncea* oocytes, and this may be relate to their deeper natural habitat and their higher level of phospholipids. Our previous works have suggested that higher levels of PE and PC are found in early stage coral oocytes and create a higher surface viscosity that increases membrane fluidity [18].The present study also showed that LPTT value of early stage oocytes was significantly lower than that of late stage oocytes. In fact, the oocytes membranes in the three different gorgonian species demonstrated different LPTTs at different developmental stages, which may be due to changes in the phospholipid composition of their plasma membranes that occur over the course of their maturation. This current study of the LPT behavior of gorgonian coral oocytes demonstrates that the LPTT is a critical parameter to assess in gauging whether or not a coral can be successfully cryopreserved.

## Materials and Methods

Ethics Statement: these corals are not regulated under Taiwanese law and the coral collection was approved by Kenting National Park.

### Collection of *J. Fragilis, J. Juncea,* and *E. Robusta*

Three gorgonian corals; *J. fragilis, J. juncea and E. robusta* were collected by SCUBA divers from reefs within Nanwan Bay, Kenting National Park, Taiwan (21°56′N, 120°44′E) during between July and September 2013. The *J. fragilis* colonies were located on the seaward reef slopes at a depth range of 3–5 m, whilst *J. juncea and E. robusta* communities were collected from below 20 m. Three specimens of each species were cut into ∼60-cm branches with surgical scissors. After fragmentation, the coral branches were transporter in 200 L containers filled with seawater and taken to the Coral Husbandry Center of Taiwan’s National Museum of Marine Biology and Aquarium (NMMBA). Once at NMMBA, the coral branches were transporters to flow-through seawater tanks (4 tons) maintained at 25°C for further processing.

### Oocyte Isolation

Coenchyme tissues from coral branches were excised with sterile scalpels and transferred directly to 6-well tissue culture dishes containing 2 ml filtered (0.4 μm) seawater (FSW; 35 psu). Oocytes were mechanically separated from the coenchyme tissue with sterile forceps followed by aspiration with a pipette under a dissecting microscope (Olympus, SZ51, Japan). The oocytes were washed three times with FSW and then stored in FSW at 25°C for further processing. The developmental stages of the gorgonian oocytes were determined based on the classification scheme of Lin et al. 2011 [1]. Briefly, oocyte diameters were measured under the microscope with an ocular micrometer (Olympus, C31, Japan). The oocytes in the early stage were in the range of 50–200 μm, whereas late stage oocytes ranged from 200 to 350 μm.

### Effect of Chilling on the LPT of Coral Oocytes

The effect of chilling on the LPT of coral oocytes at different developmental stages was investigated. Oocytes in each test tube (1.5 mL) were placed in a low temperature bath (Dry bath, CB-1502, Medclub Scientific CO., LTD., Taiwan) at 5°C, and the oocytes were allowed to chill for 0, 48, 96, and 144 h. After chilling, oocytes were warmed in a water bath at 25°C and washed twice in with FSW. Control oocytes were kept in 35 psu FSW at 25°C. The total number of oocytes in each test tube was quantified under the light microscope (Olympus, C31, Japan).

### Measurement of Membrane Phase Transition

The membrane phase transition of oocytes was determined with a Bruker-Tensor 27 FTIR spectrometer connected to a Bruker FTIR Hyperion 2000 microscope (Ettlingen, D-76275, Germany). The mercury-cadmium telluride detector within the microscope was cooled down with liquid nitrogen before measurement of the samples. Oocytes were positioned on a silicon wafer which was then placed in a temperature controlled IR stage (Linkam, FTIR 600, United Kingdom) and measured under the reflectance mode under the microscope. The sample temperature was controlled with a thermoelectric cooler at the range of 30 to 0°C at 2°C intervals and to within 0.1°C at each temperature by microprocessor – controlled cooling system (Linkam, PE95/T95, UK) equipped with temperature control and capture software (Linkam, Linksys 32, United Kingdom). At each temperature, a total of 128 scans at a resolution of 4 cm^−1^ were collected for each oocyte.

The properties of the membranes were determined from the temperature influence on the methylene (CH_2_) asymmetric stretching vibration frequency, which is particularly sensitive to the conformational order of the lipid acyl chain; briefly, there is a lower wave number in gel phase membranes compared to those of the more fluid lamellar phase. All the spectral analyses, operation qualification test for improving the quality of the images, and reference and sample measurements for attenuated total reflectance calculations following the FTIR studies were performed with OPUS spectroscopy software (Bruker, Version 7.0, Germany) on a PC computer. The membrane LPTT curve from liquid crystalline to the gel phase was determined by statistical analysis.

### Statistical Analysis

Each treatment in the experiment contained three replicates, and experiments were repeated at least three times. The data were analyzed with SPSS software (Version 17.0; SPSS Inc., Chicago, IL, USA). The data were checked for normal distribution based on a modification of the Kolmogorov-Smirnov one-sample test and homogeneity of variance was determined with Levene’s test. In all statistical tests used, *p<*0.05 was considered to be significant. Results are presented as means ± SEM.
